# Iron Oxide-Au Magneto-Plasmonic Heterostructures: Advances in Their Eco-Friendly Synthesis

**DOI:** 10.3390/ma15197036

**Published:** 2022-10-10

**Authors:** Marta Miola, Cristina Multari, Enrica Vernè

**Affiliations:** Department of Applied Science and Technology, Politecnico di Torino, Corso Duca degli Abruzzi 24, 10129 Torino, Italy

**Keywords:** magnetic nanoparticles, gold nanoparticles, magneto-plasmonic heterostructures, green-synthesis

## Abstract

In recent years, nanotechnologies have attracted considerable interest, especially in the biomedical field. Among the most investigated particles, magnetic based on iron oxides and Au nanoparticles gained huge interest for their magnetic and plasmonic properties, respectively. These nanoparticles are usually produced starting from processes and reagents that can be the cause of potential human health and environmental concerns. For this reason, there is a need to develop simple, green, low-cost, and non-toxic synthesis methods and reagents. This review aims at providing an overview of the most recently developed processes to produce iron oxide magnetic nanoparticles, Au nanoparticles, and their magneto-plasmonic heterostructures using eco-friendly approaches, focusing the attention on the microorganisms and plant-assisted syntheses and showing the first results of the development of magneto-plasmonic heterostructures.

## 1. Introduction

In the last few decades, research in the medical sector has been enriched by new approaches based on nanotechnology [1,2], with a particular focus on innovative systems for imaging, drug delivery, cancer therapy, and theranostics [3,4].

In particular, theranostics comprises a combination of diagnosis, therapy, and follow-up of a disease, which takes advantage of the capacity of nanosystems to have both imaging and therapeutic functionalities. Nanoparticles (NPs) have attracted great interest because of the possibility of using them in the field of tumor therapy. They have emerged as key players in modern medicine thanks to their numerous applications in tumor targeting, contrast agents in magnetic resonance imaging, and hyperthermia, as well as in the delivery of therapeutic payloads at tumor sites [5,6]. Both organic and inorganic NPs and their combinations have been proposed as nanocarriers for many therapeutic strategies, providing noninvasive visualization of the targeted tumor (imaging) and carrying multiple theranostic agents. 

In particular, the use of magnetic NPs (MNPs) in nanomedicine offers several advantages, both in therapy and in in vitro/in vivo diagnostics [7,8,9], due to the possibility of tailoring their properties by controlling the shape, size, microstructure, synthesis method, stability and surface properties. MNPs are known for their capacity to be directed near the tumor site by magnetic driving without retaining any magnetism after the removal of the magnetic field. They can also be functionalized to carry drugs, nucleic acids, monoclonal antibodies and, viral vectors and are used for diagnostic assays and for the generation of local hyperthermia for tumor therapy. 

The potential applications of MNPs in the biomedical field can be further enhanced by their decoration with ultra-small metal nanoparticles, joining the magnetic properties of the core with the optical properties of metal nanoparticles [10,11]. For example, gold NPs (Au NPs) have been the object of several studies due to their surface plasmon resonance (SPR) absorption at visible or near-infrared wavelengths, which make them useful, for example, as photosensitizers in photothermal therapy [12,13]. In fact, they can be externally activated and remote controlled by using a light stimulus. Photothermal therapy mediated by Au NPs is particularly attractive for cancer treatment: the intracellular uptake of Au NPs, which upon irradiation convert absorbed light into thermal energy, produces intracellular heating and disrupts cancer cells, due to their increased heat sensitivity.

The combination of MNPs and Au NPs has been widely exploited to create hybrid nanoplatforms (magneto-plasmonic heterostructures) with several potential applications in biomedicine, such as magneto-photothermal therapy of cancer [14,15]. Usually, the synthesis methods for such hybrid nanoplatforms combine different wet chemistry steps, aimed to induce the nucleation and growth of MNPs and Au NPs, starting from soluble precursors, sometimes using toxic chemicals that can be the cause of potential health and environmental concerns or exposure risks for the operators [16]. The awareness of the need for developing green synthesis approaches to avoid the problems related to synthesis procedures has therefore stimulated a wide range of simple, low-cost and non-toxic approaches [17,18,19]. 

Given their relevance in theranostics and their collocation in an as yet incomplete area of the study of their production procedures, iron-oxide, and Au nanoparticles, as well as iron-oxide/Au magneto-plasmonic heterostructures, will be the subject of the present review, with a special focus on original green synthesis approaches. 

For this research, reviews and papers from the most important publishers were consulted, mainly considering publications in the last 15 years, by searching specific keywords, such as magnetic nanoparticles, Au nanoparticles, green/eco-friendly synthesis, and magneto-plasmonic heterostructures/nanostructures. 

This review is divided into three parts: the first one (Section 2) is a brief overview of the magneto-plasmonic heterostructures that can be developed, the second part (Section 3), gives the reader a concise review of the main applications of magneto-plasmonic heterostructures in the biomedical field, the third part (Section 4) is dedicated to the description of eco-friendly approaches to developing magnetic (based on iron-oxides) and plasmonic (Au) nanoparticles, and to the illustration of the first green attempts to the design of magneto-plasmonic heterostructures. The review concludes by providing some indications for prospective research and future challenges in the field.

## 2. Overview of Magneto-Plasmonic Heterostructures

The magneto-plasmonic nanostructures based on iron oxides and gold nanoparticles are mainly divided into two categories: the FAuNSs (Fe_3_-xO_4_-Au nanostructures), which consist in a magnetic core on whose surface Au NPs or Au coating are attached or the AuFNSs (Au-Fe_3_-xO_4_ nanostructures) constituted by a single or multiple Au cores, surrounded by a Fe_3_−xO_4_ coating [14]. The magnetic properties of nanostructures are determined by the synthesis method, the chemical composition of the magnetic fraction (e.g., pure magnetite nanoparticles or a combination of magnetite/maghemite nanoparticles), their size and their morphology/architecture (e.g., single core, multicore, clusters) [20,21,22]. Regarding the properties of plasmonic nanoparticles, they can be modulated by tailoring the shape of the gold component [14,15,23,24], from a continuous thin layer (spherical or not spherical) to small Au particles.

The main features of FAuNSs are the aptitude to be easily conjugated with different biological moieties due to Au NPs surface ability to be functionalized with thiol groups, the affinity of Au surface for different electron-donating groups, several hydrophilic ligands and polymers [25,26], enhancing the functionality of magnetic NPs. Moreover, the gold NPs/coatings protect the nanostructures from chemical oxidation, confer them stability by preventing the nanostructures agglomeration and flocculation, and improve their biocompatibility. With regard to AuFNSs, these structures normally have important magnetic properties, due to the spin alignment at the gold/iron oxide interface and the dipolar interactions between one AuFNS and another.

Both FAuNSs and AuFNSs can be mainly divided into different categories on the basis of their structures:

(a) Core-satellites: in FAuNSs iron-oxide nanoparticles, single or multicore, can be surrounded by small Au NPs grafted on a magnetic particles surface, or an inorganic (e-g, silica) or organic shell (including small organic molecules or surfactants, polymers, and biomolecules) can be inserted between magnetic and plasmonic structures. Alternatively, a nanodumbell structure can be obtained. In AuFNSs, a gold nanoparticle is surrounded by several magnetic small particles [10,27];
(b) Spherical core-shell: in this case, a magnetic core (single or multicore) is coated with a thin layer of gold or gold and an intermediate layer (FAuNSs); in contrast, a single gold nanoparticle can be coated by a magnetic shell, which possesses high magnetization [28,29,30];
(c) Non-spherical core-shell: both FANSs and AFNSs can be also obtained by coating non-spherical Au/iron oxide non-spherical particles (e.g., rods, stars or flowers). The non-spherical shape can affect the optical and magnetic properties of the nanostructures. For example, Au nanorods possess an absorption peak shifted to the NIR region, compared to Au nanoparticles [31,32,33];
(d) Hollow structures: they are mainly composed of a core of iron oxide and a shell of gold. Between the magnetic core and the shell there is an empty space, which confers a very high specific area and reactivity to the nanostructures. Moreover, these hollow structures possess unique optical and magnetic properties and the hollow space can be used to load drugs [34,35].

**Figure 1:**
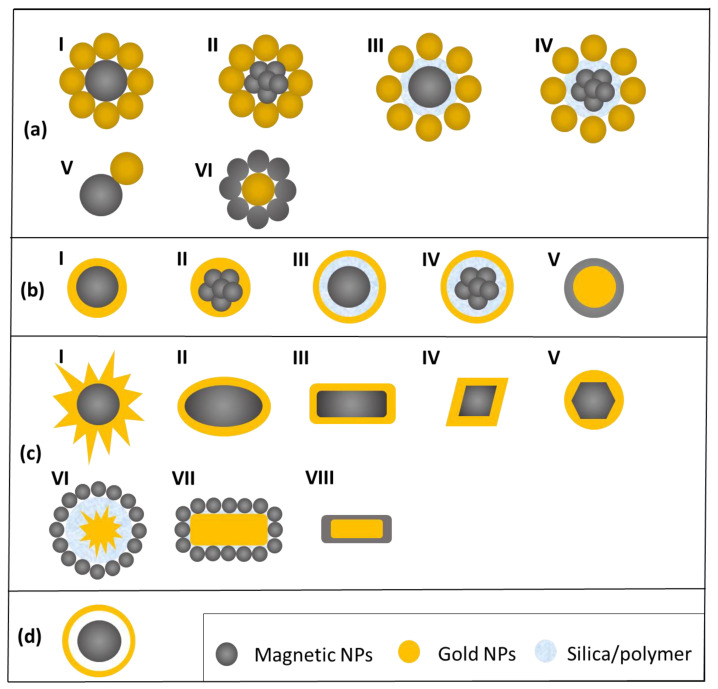
Examples of iron oxide-gold nanostructures: (**a**) core-satellites with different structures (I–VI), (**b**) spherical core-shell with different structures (I–V), (**c**) non-spherical core-shell with different structures (I–VIII), (**d**) hollow structures.

Figure 1 shows a scheme of the possible magneto-plasmonic structures (FAuNSs and AuFNSs).

## 3. Main Applications of Magneto-Plasmonic Heterostructures in the Biomedical Field

### 3.1. Photothermal Therapy (PTT)

**Figure 2:**
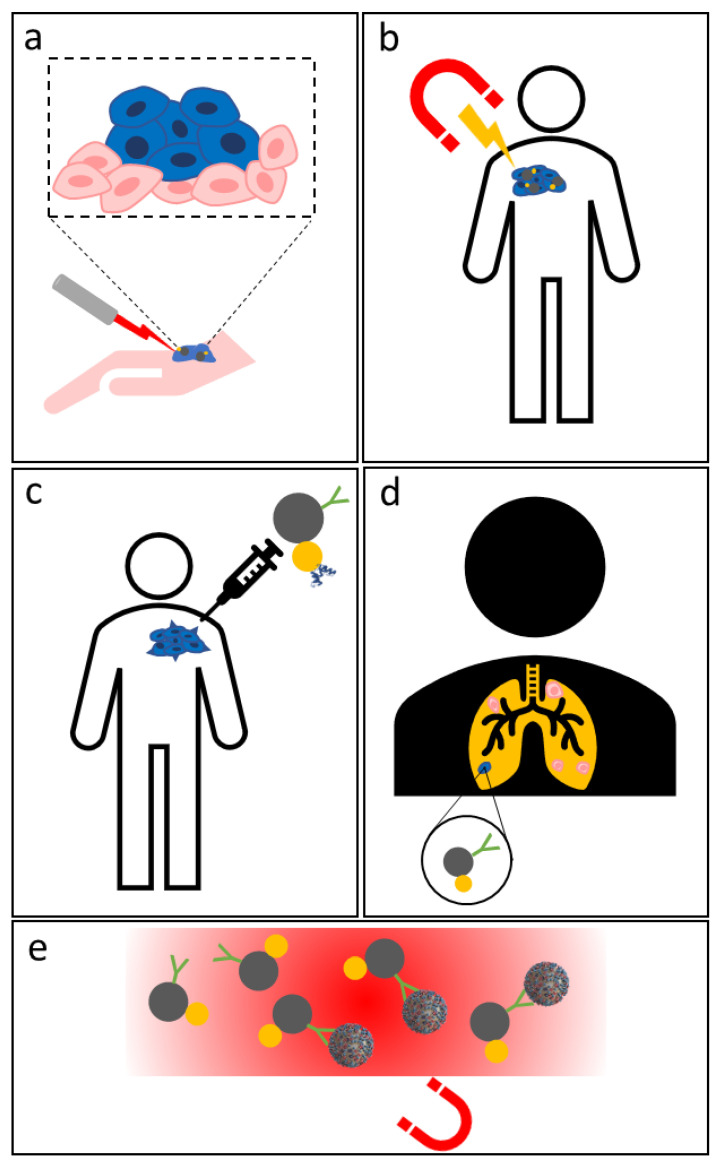
Schematization of photothermal therapy application (**a**), magnetic hyperthermia (**b**), drug delivery and targeting (**c**), bioimaging (**d**) and SERS application, detection and separation (**e**).

PTT is a therapy that consists in exploiting light radiation in the visible (Vis) or near-infrared (NIR) region to produce heat in a specific target region of the body, in order to induce cancer cells apoptosis and kill them (Figure 2a). Due to the well-known SPR effect, gold nanoparticles can be irradiated with laser light having a specific wavelength, transforming the radiation into thermal energy, thus producing heat, which induces tumoral cells in apoptosis [36,37]. Au NPs are widely investigated since they possess million times higher absorption capacity than conventional dye molecules, they are photostable, biocompatible, and have a high conversion efficiency of the absorbed light into heat. Their great conversion efficiency allows some of the most common problems of conventional organic dyes to be overcome, such as the photobleaching phenomenon, to which organic dyes are subjected, and the low absorption efficiency [38]. 

One of the main advantages of PTT is the use of light as an external stimulus, since it is easy to regulate, focus, and remotely control using a pulsed or continued wave laser. The employment of laser light as a source and the connected benefits allow a better tumor-targeted treatment, preserving healthy tissues from damage since tumoral cells and tissues have higher heat sensitivity in comparison with healthy ones [39,40]. The heat produced by nanostructures, which should be between 43 °C and 46 °C, alters the functions of the tumoral cells, causing their destruction through apoptosis. Furthermore, the tumoral tissue is poorly vascularized and thus the temperature rises higher than in the surrounding well-vascularized or normal tissues. Hyperthermia can also kill hypoxic cells that are radiation resistant, both directly, as a function of the reached temperature and the heating period, or indirectly, increasing blood flow and thus improving tissue oxygenation, and radio-sensitizing via DNA repair inhibition. Moreover, Au NPs can also be functionalized with specific target ligands useful for focalizing the treatment at the tumor site [41].

The coupling of Au nanoparticles with iron oxide nanoparticles allows the PPT to be combined with the magnetic features of iron oxide NPs. Moreover, recent studies reported that the use of a magnetic field can cause a reversible aggregation of nanostructures with an enhancement of the surface-enhanced Raman spectroscopy signal (SERS), a shift of their plasmonic absorbance, and an increase in the efficiency of the NIR-induced PTT/photothermal effect [42,43,44,45].

### 3.2. Magnetic Hyperthermia

Magnetic iron oxide nanoparticles, and in particular Fe_3_O_4_ NPs, have been widely investigated and applied in biomedical applications, due to their biocompatibility and the possibility of functionalizing them or coated them with different chemical and biological species [46,47].

Magnetic nanoparticles, when subjected to an alternating electromagnetic field, possess the ability to convert electromagnetic energy into thermal energy due to magnetic losses, and thus they can be used in the hyperthermia treatment of tumors [48,49] (Figure 2b).

Hyperthermia can directly kill cells, leading to a loss of cellular homeostasis by protein denaturation and aggregation and the increase in reactive oxygen species [50]. Several studies have also shown that hyperthermia treatment enhances the effects of chemotherapy and radiotherapy, reducing their side effects [51,52,53,54,55,56,57,58,59]. Moreover, it has been demonstrated that hyperthermia can kill hypoxic cells and cells that are in the S phase, which are resistant to radiotherapy [56].

Their ability to release heat depends on the particles size, shape, and coercivity as well as on the power and frequency of the used alternating magnetic field [60]. Also, the aggregation state can influence the nanoparticles’ ability to release heat, as reported in [61]. Finally, the use of the magnetic field as a stimulus allows the use of hyperthermia, even in deep tissues [48,62].

Magnetic nanoparticles have been also proposed as a therapeutic system in conjunction with hyperthermia. The application of an alternating magnetic field can favor the release of drug molecules attached to the magnetic NPs surface by a linker. The developed heat can cause the degradation of the linker molecule, promoting the release of the drug, and simultaneously kill cancer cells that are sensitive to heat [63]. By tailoring the intensity and frequency of the applied electromagnetic field, it is possible to modulate the drug release kinetics and combine it with hyperthermia.

The opportunity of creating magneto-plasmonic heterostructures allows combining the magnetic hyperthermia and the unique properties of plasmonic particles (e.g., SERS application, easy functionalization), thus designing technological nanomaterials for diagnosis and therapy.

### 3.3. Drug Delivery and Targeting

Drug delivery consists in the distribution of substances and drugs in a specific area of the human body affected by a disease or a tumor. This process is more specific and effective than systemic administration and allows side effects on the healthy tissues to be limited, and to reduce the necessary dosage.

The process basically consists in the injection of magneto-plasmonic nanoparticles in the body area affected by a tumor, or alternatively, the nanostructures can be intravascular injected and magnetically guided at the tumor site. Once reached, the desired location, the drug/bioactive agent can be released in a controlled manner using an external magnetic field or through changes in physiological conditions such as pH, temperature, or osmolality [64]. Ideally, a drug delivery system should contain the maximum drug load and release it at the desired site with detailed kinetics in function of the envisaged disease. For this reason, the dimensions, the charge, and the surface chemistry of the nanoparticles are particularly important features in this application as they strongly influence the circulation time in the body through the blood vessels. Generally, the optimal diameter range for this application is around between 10 and 100 nm; in fact, smaller nanostructures can be easily expelled through kidney cleansing, while the upper limit is not well defined, and it can be set according to the type of tumor into which the nanoparticles must penetrate, and the administration route.

Magneto-plasmonic nanoparticles adopted in drug delivery and targeting often consist in hollow nanostructures, due to the chance of physically entrapping drugs/bioactive agents in their hollow interior [64]. Differently, both magnetic and gold NPs could be functionalized with different targeting molecules and act as drug delivery systems in order to deliver and locally release therapeutic substances to destroy specific cells (Figure 2c). Magnetic nanoparticles used as a controlled delivery system are often functionalized or coated with biocompatible responsive polymers, able to encapsulate large amounts of drugs, and silica, which shows many -OH groups useful for further functionalization, or mesoporous silica, which presents a highly controlled porosity useful to adsorb drugs [65]. Even gold nanoparticles can easily be functionalized with high-affinity moieties (e.g., ligands) able to detect cells or tissues in a selective way; they can accumulate in diseased tissues and release their payload directly on the target site, thus reducing the drug dose administered, and limiting undesirable absorption into healthy tissue and consequent side effects [66]. One of the advantages of using Au NPs as drug carriers is provided by their simple synthesis methods, involving the use and functionalization of specific chemical species on the surface of the Au NPs. These molecules form a shell that covers and stabilizes the particles and at the same time provides binding sites (such as free–SH groups) to which various other payloads can be easily attached.

Therefore, the functionalization of magneto-plasmonic heterostructures can both allow the systemic distribution of drugs by reducing their related side effects and decrease the required dose for the treatment.

### 3.4. Bioimaging

Superparamagnetic nanocrystals of iron oxides can be used as contrast agents in magnetic resonance imaging (MRI), a non-invasive imaging technique used in the medical field to discriminate different tissues on the basis of their biochemical composition [67]. MRI is based on the nuclear magnetic resonance of the protons of the water present in the body. Tumors and diseased cells can be detected due to the different return rate to the original magnetization situation by protons placed in different tissues; this effect is used to create a contrast between different types of tissue in the body. By using magnetic nanoparticles, the resolution of MRI images greatly increases, allowing the identification of diseases or tumoral areas, which cannot be detected by other techniques.

In particular, iron oxide-based magnetic particles (eventually coated by a gold shell), thanks to their ability to significantly reduce transverse relaxation times, are studied and approved by the FDA as T_2_ contrast agents for magnetic resonance imaging [68,69]. Moreover, due to their physiochemical properties, iron-oxide nanoparticles are recently investigated as T1 contrast agents; preclinical studies using superparamagnetic iron oxides as an alternative to Gd^3+^-based contrast agents are ongoing [70,71].

Recently, the use of magnetic nanoparticles in magnetic particle imaging (MPI) has also attracted considerable attention [67,72]; this modality is currently used in the preclinical field and does not use nanoparticles as a contrast agent but as a tracer. In MPI, the magnetic particles, in particular those based on iron oxide, are subjected to an oscillating magnetic field generating a signal derived from their non-linear magnetization [73]. The technique can provide four-dimensional information on the spatial distribution of NPs and quantifies their concentration with resolution; several studies have been carried out at a pre-clinical level for different biomedical applications, including cancer tracing and the MPI guidance of magnetic fluid hyperthermia [72].

With regard to Au nanoparticles, their specific optical properties from the wavelengths of visible light to NIR, thanks to the high electron density of gold, can be used as nanosources in optical imaging, or in optical coherence tomography, computed tomography, dark field microscopy and photoacoustic imaging [14].

Due to the high scattering properties and the high photostability, if compared with other dyes, Au NPs have emerged as an extremely promising tool for cellular imaging and monitoring. The scattering ability of Au NPs depends on their size and shape; different studies report a suitable Au NPs size range between 20 and 100 nm [74]; their strong emission power makes them easily detected and so they are a promising tool for cancer cell imaging (Figure 2d). 

By combining the characteristics of plasmonic and magnetic nanoparticles, more precise diagnoses can be obtained, as the integration of these nanostructures allows the respective strengths to be combined and eliminates the critical aspects, such as the low sensitivity of the magnetic resonance, which is compensated for by the plasmonic NPs.

### 3.5. SERS Application, Detection and Separation

For the selection and separation of biological and chemical molecules, the most commonly used method is Surface-Enhanced Raman Spectroscopy (SERS). As evidenced by several studies [75,76,77,78], the use of plasmonic nanoparticles, in particular Au NPs for their high chemical stability and their SPR support in the visible region, can significantly improve the SERS signal both in vitro and in vivo.

The combination of plasmonic nanoparticles with magnetic nanoparticles allows for the concentration and the potential separation of analytes, achieving high sensitivity targeting. The concentration and aggregation of nanostructures also allow for the creation of hot spots, which increase the intensity of the SERS and thus promote sensitivity. Moreover, the magnetic component can be exploited for immobilizing the nanoparticles on a solid substrate.

For these reasons, magneto-plasmonic heterostructures are often investigated for the detection and isolation of low concentrated small molecules, such as nucleic acids or proteins, as reported in [79,80,81,82], or for the detection and isolation of pathogens, such as bacteria and virus, both directly or indirectly, using specific antibodies [83,84,85,86] (Figure 2e).

## 4. Green Synthesis Methods of Magneto-Plasmonic Heterostructures

The syntheses of iron oxide or gold nanoparticles, as well as the development of hybrid iron oxide–gold heterostructures are often hazardous and expensive, involve the use of harmful chemicals and sometimes toxic reducing and dispersing agents that lead to biological risks and the formation of environmentally pollutant coproducts [87,88].

Several methods for the synthesis of magnetic and plasmonic nanoparticles, as well as magneto-plasmonic heterostructures, have been reported in the literature, including co-precipitation technique, thermal decomposition, solvothermal method, Brust’s method and flame aerosol synthesis [89,90]. However, these chemical and physical syntheses of nanostructures present numerous issues, mainly due to the use of toxic chemicals during the process steps, which could limit their use in medical field. For these reasons, there is an essential need to develop easier, environmentally friendly and cost-effective procedures for nanoparticles synthesis suitable for large scale production.

In recent years, the biological synthesis of nanoparticles and nanostructures has been proposed as a new approach and has attracted great attention [91,92,93]. This new ecological approach for the synthesis of nanostructures presents various advantages such as simplicity, cost-effectiveness, compatibility for biomedical and pharmaceutical applications, as well as large-scale commercial production. A promising approach for achieving this goal is to exploit the wide range of biological resources. In fact, over the last few years, plants, algae, fungi, bacteria and viruses have been used for metallic NPs production with low-cost, low-energy dissipation and non-toxic method [93].

The synthesis and assembly of nanostructures would benefit from the development of clean, non-toxic and environmentally friendly technologies that go under the name of “Green Chemistry”. Green chemistry aims to use less or avoid using hazardous substances in a chemical process. In fact, green chemistry is a movement to find alternatives to the use of hazardous chemicals such as feedstock, reagents, solvents, products, and byproducts in the production processes. Moreover, it is concerned with the sustainability of using raw materials and energy sources for manufacturing [93]. 

The use of Green Chemistry in nanosciences allows high precision in the synthesis and low production of waste to be achieved, which are enormous benefits in production on a commercial scale, for society and the environment [94]. The use of low-cost chemicals, non-toxic and biodegradable solvents is essential for the synthesis of non-dangerous materials for both the environment and humans. Several metal and non-metal nanoparticles can be obtained by the biochemical processes in biological agents, by means of rapid procedures in comparison with chemical routes. Moreover, some of the eco-friendly components can be used as both reducing, capping, and stabilizing agents, limiting the use of further agents and accelerating the synthesis of the nanostructures [95]. The reducing agent, the reaction medium, and the stabilizing agent are the three key factors for the synthesis and stability of nanoparticles also in biological synthesis [95,96]. In particular, during the synthesis of nanoparticles by means of microorganisms and plant extracts, several process parameters should be carefully controlled to obtain stable and dispersed nanoparticles, such as the temperature, the pH of the medium, the reaction time, and the mixing ratio.

Although the complete synthesis of magneto-plasmonic nanostructures through green processes is still poorly investigated, several studies have focused on the biological and eco-friendly synthesis of magnetic or plasmonic particles. The following paragraphs summarize the most important concepts concerning the synthesis of magnetic and plasmonic nanoparticles (Au) using microorganisms, plants extract/derivatives and other green materials (e.g., enzymes, vitamins, polysaccharides, and amino acids, etc.).

### 4.1. Microorganism-Assisted Synthesis of Iron Oxide and Au NPs

A broad range of microorganisms, such as bacteria, cyanobacteria, fungi, mycetes, yeast, and algae, have been investigated as potential nanofactories for the production of inorganic nanoparticles, avoiding the use of toxic reactants and the requirement of high energy. The precise mechanisms by which bacteria synthesize nanoparticles are not yet fully understood. However, it seems that the pathways of defense response and the different reductase enzymes contained in the bacterial cell wall (e.g., NADPH-dependent reductase, cytochrome oxidase, phosphoadenosine phosphosulfate reductase, sulfite reductase, fibrinolytic enzymes, and hydrogenase) are responsible for the synthesis of inorganic nanoparticles, such as gold, iron, silver, lead, titanium oxide and iron oxide [95,96,97]. Table 1 summarizes the main investigative studies concerning the microorganism-assisted synthesis of iron oxide-based and Au nanoparticles. The syntheses of NPs mediated by microorganisms, besides being eco-friendly, are also cost-effective, due to the abundance of microorganisms in different environments. Moreover, the syntheses are easily controllable from the chemical and physical points of view. The microorganism-assisted synthesis can be performed both intracellular or extracellular; in the intracellular process, the positive precursor ions are transported inside the cell wall, which is negatively charged. Ions then diffuse through the cell wall by electrostatic attraction and are reduced by the enzymes present in the cell walls. In the extracellular mechanism, metal ions are trapped on the surface of the microorganisms, which can exchange electrons with extracellular environments, and they are reduced in the presence of some enzymes (e.g., nitrate reductase or hydroquinone).

Among the different nanoparticles obtained using microorganisms, monodispersed iron-oxide-based magnetic NPs, which possess high purity, little crystalline defects, and narrow size, have been also developed [98,99,100,122]. Iron-oxide NPs can be produced extracellularly (biologically induced biomineralization) or intracellularly (biologically controlled biomineralization); the second route allows more ordered crystalline nanoparticles to be obtained, having a narrow size distribution and controlled morphology.

**Figure 3:**
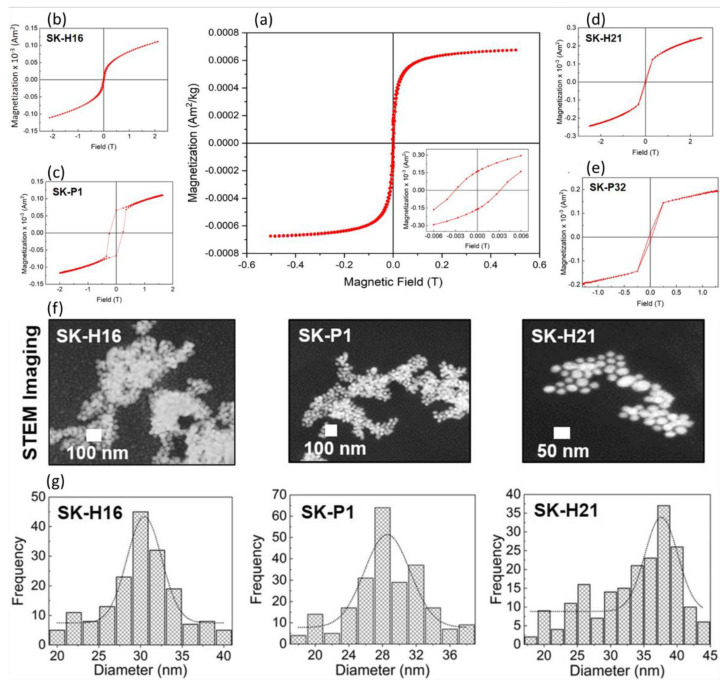
(**a**) Magnetization curve at 300 K of the magnetic nanoparticles synthetized by *Pseudomonas aeruginosa*; (**b**–**e**) Magnetic analysis performed with four bacterial cells evidencing that nanoparticles had a small remanence and coercive fields; (**f**) Transmission electron microscopy images showing the pseudo spherical shape f three isolates; (**g**) The magnetic nanoparticles diameter (size) distribution obtained from the STEM images. Adapted with permission (Creative Commons CC BY license) from [100].

As an example, the extracellular method was adopted by Majeed et al., who developed well-dispersed Fe_3_O_4_ nanoparticles (20–30 nm in diameter) using *Proteus vulgaris* [98]. The nanoparticles obtained showed a good antibacterial effect towards methicillin-resistant *Staphylococcus aureus* (MRSA) and antioxidant activity. Moreover, the NPs demonstrated high cytotoxicity against U87 MG—glioblastoma cancer cells and less toxicity towards the normal L-132 cells, proving that microbiologically synthesized nanoparticles are safe and eco-friendly. In another study, intracellular, cost-effective, and monodispersed (due to the protein sheaths that acted as naturally surface stabilizing agents) magnetite NPs were produced using different clinically isolated *Pseudomonas aeruginosa* strains (named SKP1, SKP32, SKH16, and SKH21), a magnetotactic bacterium [100]. The results obtained (Figure 3) evidenced the formation of biocompatible, well-dispersed magnetic nanoparticles, ranging in size from 35 to 50 nm in diameter. Among other bacteria, investigated as magnetic nanoparticles reducing agents, we can mention: *Thermoanaerobacter ethanolicus*, *Magnetospirillium magnetotacticum*, and *Shewanella* sps. [100,101]. The features of the obtained nanoparticles, as well as the type of bacterium, depend on different parameters, such as the pH of the culture medium, environmental parameters, the temperature, and the redox potential. Moreover, the use of magnetotactic bacteria, cells able to intracellularly mineralize nano-sized inorganic magnetic crystals [123], allows other chemical reagents not to be used, and therefore it is, among the mentioned approaches, a completely green method.

Fungi have also been used for the production of iron oxide nanoparticles [58,59]. They can easily be handled in the laboratory, possess high metal tolerance and secrete a large quantity of enzymes. Compared to bacteria, therefore, they are capable of producing nanoparticles on a larger scale [124]. As an example, Bharde et al. synthesized magnetite nanoparticles using both *Fusarium oxysporum* and *Verticillium* sp. starting from ferric and ferrous salts [102] in acidic conditions. The use of *Fusarium oxysporum* allowed pseudo-spherical magnetite NPs to be synthesized in the size range from 20 to 50 nm, while cubo-octahedrally shaped magnetite NPs between 100 and 400 nm were obtained using *Verticillium* sp. Both the nanoparticles obtained exhibited signatures of a ferrimagnetic transition with a negligible amount of spontaneous magnetization at room temperature. The protein analysis suggested that the cationic proteins secreted by the fungi are responsible for the cellular hydrolysis of the anionic iron complexes and the capping of magnetite nanoparticles. Thus, the ability of fungi to hydrolyze iron metal demonstrated the huge potential for the development of eco-friendly and large-scale syntheses of magnetic iron-oxide-based nanoparticles. In another study [103], spherical Fe_3_O_4_ nanoparticles together with pure Fe nanoparticles (between 9 and 50 nm) were prepared using the fungus *Aspergillus niger* and supercritical condition methods. The particles obtained possessed a saturation magnetization of 112 (Fe) and 68 emu/g (Fe_3_O_4_), allowing their use in different biomedical applications such as hyperthermia and contrast agent.

Plasmonic NPs, such as Au and Ag, have been also produced using microorganisms both intracellularly and extracellularly [125,126,127]. Several bacteria, such as *Caldicellulosiruptor changbaiensis*, *Shewanella loihica*, *Micrococcus yunnanensis*, *Stenotrophomonas maltophilia*, *Actinobacterspp Rhodopseudomonas*, *P. aeruginosa*, *Bacillus*, *Escherichia coli* and *Klebsiella pneumoniae* [104,105,106,107,108,109,110,111,112,113], fungi, among them *Alternaria alternate*, *Fusarium oxysporum*, *T. koningii*, *Hormoconis resinae*, *Aureobasidium pullulans*, *Fusarium*, *Phanerochaete Chrysosporium* and *Botrytis cinerea* [114,115,116,117,118,119,128], and algae [120,121] have been investigated as a Au-nanoparticle-reducing agent. To give some examples, recently Jafari and co-authors [106] used the *Micrococcus yunnanensis J2* strain to biosynthesize Au NPs, which possessed anticancer activity towards six cancer cell lines and an antibacterial effect against both Gram-positive and negative strains. He et al. [109] used *Rhodopseudomonas capsulate* as green approach to synthesize Au NPs with different sizes and shapes, highlighting as an important parameter for the synthesis of inorganic nanoparticles mediated by bacteria the pH of the medium. By adjusting the pH medium at 7, they obtained spherical Au NPs with a diameter in the range of 10–20 nm. Concerning fungi-mediated synthesis, it is an economic, easy and scalable process to produce Au NPs, due to their bioaccumulation ability and the chance to produce a large number of extracellular enzymes responsible for the reduction process. For example, Sarkar et al., produced Au NPs by the reduction of chloroauric acid using *Alternaria alternate* [114]. The Au NPs showed different shapes, an average particle size of 12 nm and they are coated with a protein shell that enhances their stabilization. In another study, Au nanoparticles with different shapes (triangular, hexagonal, spherical, decahedral, and pyramidal) and dimensions (from 1 to 100 nm) were extracellularly obtained [119] by means of the reduction of chloroauric acid mainly due to *Botrytis cinerea* NADH-dependent reductase activity. Furthermore, as evidenced in the study by Mishra et al. [129] the use of *Penicillium rugulosum*, allowed uniform Au NPs to be synthesized much more easily than bacteria and yeast-mediated synthesis.

Algae-mediated synthesis has been also explored, due to algae’s ability to accumulate heavy metals, secrete fucoidans (polysaccharides able to reduce metal NPs), and since they are characterized by different functional groups useful for the uptake (e.g., carboxyl groups) [127].

As mentioned above, the formation of nanoparticles mediated by microorganisms in the different studies was achieved through two approaches: the interaction of metal ions with surface proteins or biomolecules present on the cell membrane and their reduction through the interaction with the extracellular content or secretome of the organisms. Both methods allow nanoparticles of fairly controlled shape and size to be obtained.

### 4.2. Plants/Derivatives Assisted Synthesis of Iron Oxide and Au NPs

Plants extracts and their derivatives have also been extensively studied and used in literature to synthesize nanoparticles. Even if a comparison is complicated, usually the synthesis of nanoparticles starting from plants/derivates extracts is an even simpler and more reproducible process than the synthesis mediated by microorganisms [130]. In particular, the synthesis of metal nanoparticles, such as Au, is extensively investigated in the literature, while the realization of metal oxide NPs, including magnetic particles based on iron oxides, is less explored, but there are still several studies on the subject.

As microorganisms, plants are easy to handle, harmless and economic. (They can also be recovered from production waste, promoting a circular economy). The plant-assisted syntheses can be carried out using distinct parts of the plant (e.g., leaves, seeds, and flowers, etc.), extracts or derivatives, rich in compounds (e.g., amino acids, polyphenols, and reducing sugars), and they often act as both reducers and stabilizers or capping agents [131,132,133,134]. Plant-mediated synthesis often concerns the preparation of an extract by washing the plant (or part of the plant) in water or other solvents, cutting it into small pieces, boiling it in distilled water, and purifying the extract with different methods, such as filtration and centrifugation. The main parameters that can affect the synthesis, the quantity of NPs obtained, as well as their shape and size, are of course the plant type, the extract/derivate concentration, proteins present in the extract, the temperature, the pH of the medium, the electrochemical potential, the NPs precursors and the incubation time. Moreover, plant-assisted synthesis has the advantage of providing faster synthesis compared to the microorganism-assisted process. 

There are two chief mechanisms involved in the synthesis of nanoparticles through the use of plants or derivatives [135]: (i) the reduction of metal ions by the surface of the proteins, due to the strong electrostatic attraction between the ions and proteins of the plants/extracts; (ii) the reduction of metal ions by various active components, among which are enzymes, vitamins, amino acids, polysaccharides, polyphenols, flavonoids and organic acids, which, in addition to forming nanoparticles, encapsulate them, preventing their agglomeration. This second hypothesis seems to be the most accepted in the literature.

Recently, iron-oxide NPs have been synthesized using different plant extracts/derivatives, such as green tea leaves, *Solanum trilobatum*, *Ziziphora tenuior*, *Persia americana*, *Abutilon indicum*, *Azadirachta indica*, *Camellia sinensisù*, *Curcumin*, *Carica papaya*, *Aloe vera*, Flaxseed, Pistachio Leaf, Carob pod, *Cynara cardunculus*, Citrus limon and many others [131,136,137]. The syntheses often lead to stable and well-dispersed nanoparticles with a controlled morphology. Moreover, by varying the synthesis parameters, particles with a specific shape (such as spherical, cubical, cylindrical, needles, and prisms, etc.) and features can be obtained [131]. Spherical magnetite-based nanoparticles, ranging from a few nm to a thousand nm have been obtained using several plant extracts. For example, Yew et al. synthesize spherical magnetite NPs with an average size of 14.7 nm using Seaweed *Kappaphycus alvarezii* as a green reducing and stabilizing agent [138]. In another study [139], Fe_3_O_4_ nanoparticles have been synthesized using leaves of *Platanus orientalis* L. plant and starting from ferric and ferrous chlorides. The average size of the nanoparticles obtained was about 35 nm. They were quasi-spherical and presented high purity and crystallinity, and can potentially be used in the biomedical field. Kanagasubbulakshmi and co-authors also used *Lagenaria siceraria* leaves extract to prepare cubic Fe_3_O_4_ NPs (20–100 nm) [140]. The synthesized NPs showed different functional groups on their surface (–OH and –COOH), which afford them hydrophilicity and they can be used for further functionalization. Moreover, they possessed antimicrobial activity towards both Gram-positive and negative strains.

**Figure 4:**
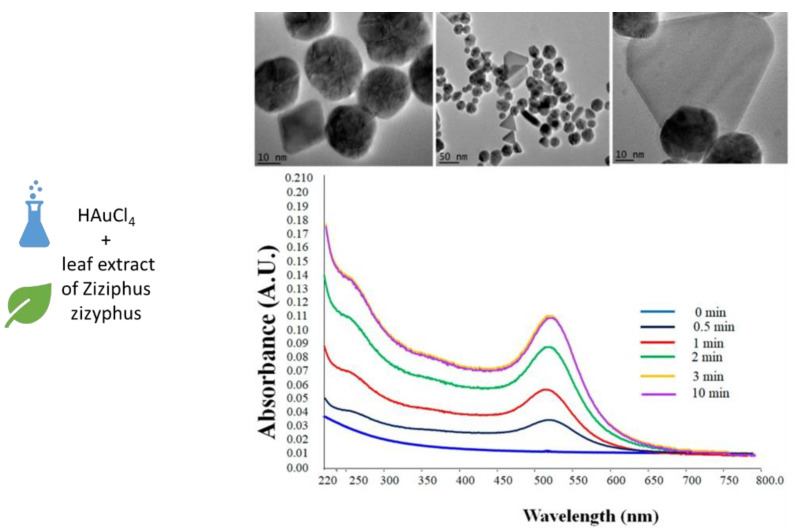
Gold nanoparticles production using leaf extract of *Ziziphus zizyphus*: UV-Vis and TEM analysis. Adapted with permission (Creative Commons CC BY license) from [146].

Obviously, also Au nanoparticles for different application fields, including the biomedical one, have been produced by means of plant-mediated synthesis [132,133,134,135,141,142,143]. The first authors to report the synthesis of gold nanoparticles using plants were Gardea-Torresdey and co-workers in 2002 [144]. As for magnetic NPs, different plant types and extracts have been investigated, among them *Pelargonium graveolens*, *Euphrasia officinalis*, *Ziziphus zizyphus*, *Indigofera tinctorial*, *Curcumin*, *Mussaenda glabrata*, *Olax scandens*, *Panax ginseng*, *Nerium oleander*, *Ananas comosus*, *Lippia citriodora*, *Mangifera indica*, *Salvia officinalis*, *Pelargonium graveolens*, *Punica granatum*, mango peel, and potato starch [135,141,142,143]. To mention some examples, one of the first optimized studies for the production of Au NPs from plants was carried out by Daizy Philip and co-authors [145]. They obtained spherical and stable Au NPs of about 20 nm in diameter in 2 min of HAuCl_4_ incubation with *Mangifera indica* extract. Moreover, they identified some soluble compounds such as flavonoids, terpenoids, and thiamine as capping ligands of Au nanoparticles. More recently, Aljabali et al. produced environmentally friendly monodispersed Au nanoparticles, starting from the leaf extract of *Ziziphus zizyphus* [146]. The nanoparticles obtained are almost spherical with an average diameter of 40–50 nm. They are biocompatible and are capped with several biomolecules, present in the plant extract, having antioxidant properties and useful for further functionalization steps (Figure 4). Spherical Au NPs have been also obtained using *Curcumin* [147] as a reducing and capping agent. The synthesized NPs exhibited antibacterial, antioxidant, and radical scavenging activities, demonstrating once again the potential of nanoparticles formed by using plants in the biomedical field. Also, non-spherical Au NPs have been obtained from plant extracts, for example Ahmad et al. synthesized hexagonal and triangular Au NPs in addition to the spherical ones, using *Trapa natans* peel extract [148]; while Geethalakshmi and co-authors developed spherical, triangular, hexagonal and cubical Au NPs (33–65 nm) with antimicrobial properties by means of the *Trianthema decandra* root [149].

Au NPs produced using mango peel extract have been reported by Yang et al. [150]; authors demonstrated that the use of mango peel extract allowed cytocompatible (up to 160 μg·mL^−1^) monodispersed Au NPs to be synthesized in the range 6.03 ± 2.77–18.01 ± 3.67 nm. They also demonstrated that the reaction rate was significantly faster than other plant extracts. 

### 4.3. Other Green Reducing Agents

A last eco-friendly approach to synthesizing nanoparticles is the direct use of green compounds (e.g., polysaccharides, natural polymers, and amino acids, etc.) to reduce metal salts [151]. Several studies reported the synthesis and stabilization of nanoparticles (including magnetic iron-oxide and gold NPs) by means of biomolecules or green active ingredients, such as heparin [152,153], glucose [154,155,156], gallic acid [157,158,159], tannic acid [159,160,161] and starch [162,163,164].

As an example, iron-oxide NPs have been produced using both tannic and gallic acid [159]; both tannins produced NPs with a size <10 nm at basic pH. The presence of the phenolic -OH groups in tannins allowed for a facile synthesis of iron-oxide NPs and supported their stabilization, avoiding the formation of aggregates.

Instead, a “clean” method for synthesizing gold and silver NPs involved the use of HAuCl_4,_ which undergo reduction with heparin and hyaluronic acid, which are reducing and stabilizers agents [152]. The NPs thus obtained show stability under physiological conditions and have important biological activities (such as anti-inflammatory and anticoagulant efficacy) demonstrated by in vivo and in vitro studies. Gorges and co-workers used glucose as a reducing agent to form Au NPs using chlorinated ions trapped in a film of amines and lipids in turn trapped on a glass substrate [154].

### 4.4. Green Synthesis of Magneto-Plasmonic Heterostructures

Combining magnetic and plasmonic properties in a single particle can bring useful synergy for several biomedical applications, such as contrast improvement in new magnetomotor imaging modalities, the concurrent capture and detection of circulating tumor cells, and molecular imaging multimodal combined with photothermal therapy. Up to now the green syntheses of iron oxides-based magnetic nanoparticles or gold plasmonic particles have been described. A completely green synthesis of composite nanostructures (heterostructures), according to the authors’ knowledge, has not yet been developed. However, in the literature, there are several studies in which at least one of the two nanoparticles or part of the synthesis process has been carried out using a green approach [11,165,166,167,168,169,170,171,172,173,174]. 

**Figure 5:**
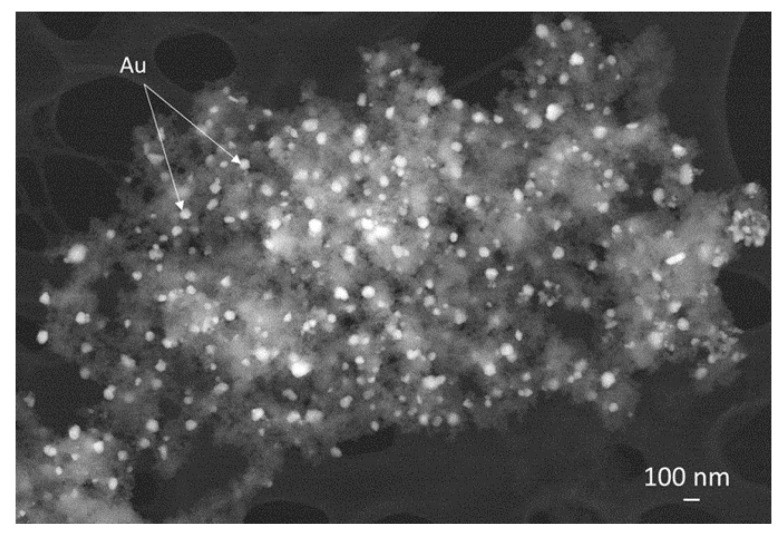
STEM analysis of magnetite nanoparticles decorated with Au nanoparticles using tannic acid as reducing agent.

Concerning the biomedical field, the authors recently proposed a green approach to develop magneto-plasmonic nanoparticles for photothermal therapy, using tannic acid or gallic acid as a green reducing agent [11,165,166]. They produced superparamagnetic iron oxide nanoparticles by co-precipitation method and subsequently, they used tannic acid to reduce in situ the Au nanoparticles on the surfaces of the magnetite obtained [11]. The hybrid nanoplatforms obtained are composed of a magnetic core and an external Au NPs decoration, forming a sort of nano-dumbbell (Figure 5). The biological characterization evidenced the nanoplatform’s ability to selectively damage cancer cells (HO-1-N-1), preserving the healthy ones (HGF) upon irradiation with a 530 nm continuous wave laser light. The authors then evaluated the influence of magnetic NPs functionalization and the different sequences of addition of tannic acid on the shape and dimensions of the composite nanoparticles [11]. They observed that the APTES functionalization of magnetic NPs can improve the grafting of Au NPs and the APTES concentration can tune the shape and dimensions of nanoplatforms, as well as the concentration of tannic acid. Moreover, in order to guide the nucleation and growth of Au NPs on magnetic NPs surface, the sequence of the addition of the Au NPs precursors and reducing agent plays a fundamental role in the shape of nanoplatforms and then in the magneto-plasmonic properties. Other authors proposed the use of tannic acid as a green reducing agent to obtain a hybrid material composed of Au NPs deposited at the surface of CoFe_2_O_4_, even if for different applications. In this case, the authors evidenced an efficient Au deposition on CoFe_2_O_4_ core functionalized with APTES [167].

Recently, Izadiyan and co-authors developed an easy method for synthesizing magnetic core-shell Fe_3_O_4_/Au nanoparticles using *Juglans regia* green husk extract [168]. The investigation of the structure, magnetic and biological properties evidenced a high saturation magnetization, a plasmon resonance of gold at about 554 nm, a cytocompatible behavior of nanoparticles up to 500 μg/mL towards non-cancer cells and the nanoparticles’ ability to inhibit the proliferation of a colorectal cancer cell line (HT-29). The magneto-plasmonic nanoparticles obtained are therefore promising devices for cancer treatment, both as drug delivery systems and as tools for photothermal therapy.

Another green approach to synthesize magnetite-gold nanoparticles has concerned the use of *Eucalyptus camaldulensis* extract as a reducing agent of gold nanoparticles [169]. Composite nanostructures of about 6–20 nm in size were synthesized by reducing Au nanoparticles in situ on magnetite surface, by subjecting the magnetic nanoparticles together with chloroauric acid to an autoclave process. The nanostructures obtained showed the presence of organic compounds with a carboxyl group on nanostructures surface, which can be exploited for the separation and purification of biomolecules by magnetic field and drug delivery.

A different environmentally friendly approach to depositing gold (or silver) shell on a magnetite core has been developed by Dizaji et al. [170]. These authors used the *Ligustrum vulgare* extract as reducing and stabilizing agents to produce magneto-plasmonic nanoparticles using a facile, green, and reproducible route. The dimensions and the magnetic properties of the obtained nanoparticles can be modulated by varying the ratio between Au precursor (HAuCl_4_) and magnetic NPs, and the quantity of plant extract. Thus, the magneto and plasmonic nanoparticles obtained are potentially useful in the biomedical field for diagnosis and therapy.

The creation of a continuous metallic shell must be carefully controlled in order to not affect the surface plasmonic resonance properties (SPR). For instance, Chin and co-authors [171] developed magneto-plasmonic core-shell nanostructures using glucose; the nanoparticles obtained showed a range between 10 and 13 nm (shell thickness about 2–3 nm); they are superparamagnetic but presented insufficient SPR properties. In another study [172], a solution containing iron salts and glucose was mixed with a second solution containing sodium silicate (which can be synthesized by extraction from rice husk ash) and gold chloride. This simple one-pot−one-step process allowed the coprecipitation of iron salts, the reduction of gold chloride, and the formation of silica resulting in a unique nanocomposite of iron oxide, silica, and gold. 

A green method for synthesizing Fe_3_O_4_/Au nanohybrid has been proposed also by Narayanan et al. [173], which used grape seed proanthocyanidin to both form magnetite NPs and reduce gold on their surface. The obtained nanohybrid showed a spherical morphology and size of about 35 nm, the magnetite core imparted superparamagnetic properties and the nanohybrid demonstrated good X-ray contrast. Moreover, the nanohybrids can be internalized by Mesenchymal Stem Cells (hMSCs) and were biocompatible up to 500 μg/mL, demonstrating once again that phytochemicals can be used to create hybrid nanoparticles for different applications in the biomedical field.

Finally, other authors proposed a facile and eco-friendly synthesis of Au–Fe_x_O_y_ nanoclusters in an aqueous solution and at room temperature [174]. The Fe_x_O_y_ nanoparticles were obtained by means of a co-precipitation process and coated with mPEG. The authors then used the amino acid L-histidine to promote the reduction of HAuCl_4_ onto colloidal Fe_3_O_4_. The nanoclusters obtained showed a size <100 nm, and useful magnetic properties which could be collected by a magnet. They were able to produce photoluminescence when excited at 488 nm and absorbed at NIR wavelengths.

Table 2 summarizes the green approaches adopted to synthesize magneto-plasmonic heterostructures.

## 5. Conclusions

This work provides an overview of the innovative green processes used to produce magnetic nanoparticles (in particular Fe_3_O_4_), Au nanoparticles and hybrid iron-oxide/Au nanostructures.

Different approaches have been developed to produce single magnetic or gold particles without resorting to using chemical reducing agents, potentially toxic to humans or the environment, while adopting plants and microorganisms, which are cost-effective and eco-friendly. These processes not only avoid the use of hazardous chemicals and toxic reducing and dispersing agents, but are often single-step, they are performed in aqueous environment, at room temperature, and they are scalable and economic from the industrial point of view. 

Concerning the synthesis of magneto/plasmonic heterostructures, some green approaches have been already explored, obtaining good results in terms of structures and magneto-optical properties. However, new efforts are necessary to reach the same level of size, morphology and crystallinity control as the conventional synthesis method and to bring the processes investigated to an industrial scale. Moreover, current green procedures concern only a part of the entire synthesis process of hybrid nanostructures. Therefore, there is still a need for extra steps to obtain a complete eco-friendly production of magneto-plasmonic heterostructures. Nevertheless, the new technologies and knowledge developed will lead to the design of completely green processes in the coming years.

## Figures and Tables

**Table 1:** Fe_3_O_4_ and Au nanoparticles recently produced using microorganisms.

Nanoparticles	Reducing Agent	Shape/Dimension	Reference
Fe_3_O_4_	*Proteus vulgaris*	Spherical/20–360 nm	[98,99]
Fe_3_O_4_	*Pseudomonas aeruginosa*	Spherical/30–50 nm	[100]
Fe_3_O_4_	*Thermoanaerobacter ethanolicus*	Octahedral/<12 nm	[101]
Fe_3_O_4_	*Magnetospirillium magnetotacticum*	Spherical/<50 nm	[101]
Fe_3_O_4_	*Fusarium oxysporum*	Pseudo-spherical/20–50 nm	[102]
Fe_3_O_4_	*Verticillium* sp.	Cubo-octahedral/100–400 nm	[102]
Fe_3_O_4_	*Aspergillus niger*	Spherical/18–50 nm	[103]
Au	*Caldicellulosiruptor changbaiensis*	Spherical/20–60 nm	[104]
Au	*Shewanella loihica*	Spherical/2–15 nm	[105]
Au	*Micrococcus yunnanensis*	Spherical/15–55 nm	[106]
Au	*Stenotrophomonas maltophilia*	Spherical/40 nm	[107]
Au	*Actinobacter* spp.	Triangular/50–60 nm	[108]
Au	*Rhodopseudomonas*	Pyramidal/10–20 nmSpherical/10–20 nm	[109,110]
Au	*P. aeruginosa*	Spherical/15–40 nm	[111]
Au	*Bacillus cerus*	Spherical, hexagonal, and octagonal/20–50 nm	[112]
Au	*Escherichia coli*	Spherical/5–130 nm	[99]
Au	*Streptomyces* sp. *VITDDK3*	Cubic/90 nm	[113]
Au	*Klebsiella pneumoniae*	Spherical/24–256 nm	[99,114]
Au	*Alternaria alternate*	Spherical, triangular, hexagonal/12–29	[115]
Au	*Fusarium oxysporum*	Spherical/20–50 nmSpherical, hexagonal, and octagonal/20–50 nm	[112,116]
Au	*T. koningii*	Spherical/10–14 nm	[117]
Au	*Hormoconis resinae*	Spherical/3–20 nm	[118]
Au	*Aureobasidium pullulans*, *Fusarium oxysporum and Fusarium*	Spherical/23–35 nm	[119]
Au	*Phanerochaete Chrysosporium*	Spherical/10–100 nm	[120]
Au	*Botrytis cinerea*	Spherical, triangular, hexagonal, decahedral, and pyramidal/1 to 100 nm.	[121]

**Table 2:** Magneto-plasmonic heterostructures produced using green approaches.

Reducing Agent	Shape/Dimension	Reference
Tannic acid	Different nanostructures/-	[11,165,166]
*Juglans regia*	Core-shell, spherical/6.08 ± 1.06 nm	[168]
*Eucalyptus camaldulensis*	Nanocomposite, core-shell/6–20 nm	[169]
*Ligustrum vulgare*	Core-shell, spherical/10.1–12.1 nm	[170]
Glucose	Core-shell, spherical/10–13 nm	[171,172]
Grape seed proanthocyanidin	Spherical/35 nm	[173]
Amino acid l-histidine	Nanoclusters/<100 nm	[174]
